# Urethrovaginal Fistula in a 5-Year-Old Girl

**DOI:** 10.1155/2015/202059

**Published:** 2015-04-12

**Authors:** Noël Coulibaly, Ibrahima Séga Sangaré

**Affiliations:** Service d'Urologie, CHU de Treichville, 01 BP 6970, Abidjan 01, Côte d'Ivoire

## Abstract

Urethral fistulas are rare in girls. They occur most of the time during trauma. The case presented here is an iatrogenic fistula. The treatment was simple and consisted of a simple dissection and suture of urethra and vagina.

## 1. Introduction

Due to abnormal communication between the urinary tract and genital tract, urogenital fistulas (UGF) may be located at different levels and occur most of the time in a traumatic context. In our daily practice, the urogenital fistulas are mainly obstetric and vesicovaginal fistulas (VVF). The urinary fistulas are rare in girls and are caused by trauma or congenital defects. The observation of an urethrogenital fistula in a 5-year-old girl poses the problem of etiology and especially its treatment that helps to preserve maximum external genitalia.

## 2. Observation

**Figure 1:**
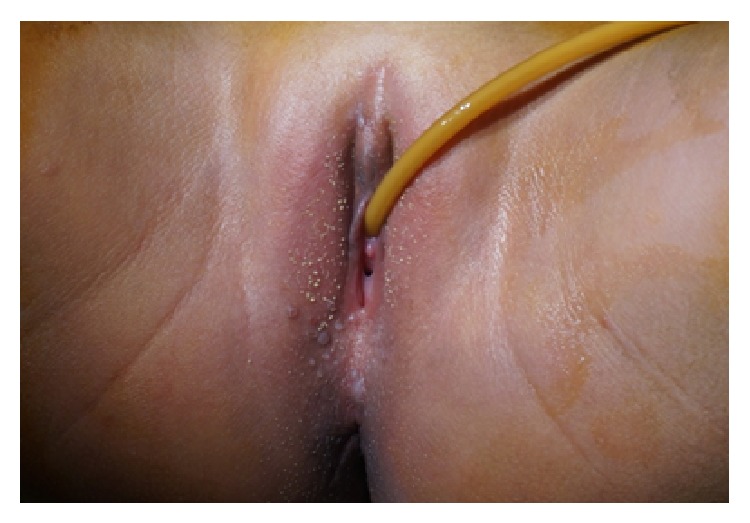
Type 1 FGM.

A 5-year-old girl, with a history of female genital mutilation (FGM) (Figure 1), was received in consultation for permanent and involuntary urine loss through the vagina. There was also a history of surgical procedure requiring an indwelling urinary catheter. Urinary loss would have occurred following this operation. Physical examination revealed a good health status with maceration lesions on external genitalia and the inner face of the thighs and Type 1 FGM. There was spontaneous and permanent loss of urine through vagina in orthostatism.

**Figure 2:**
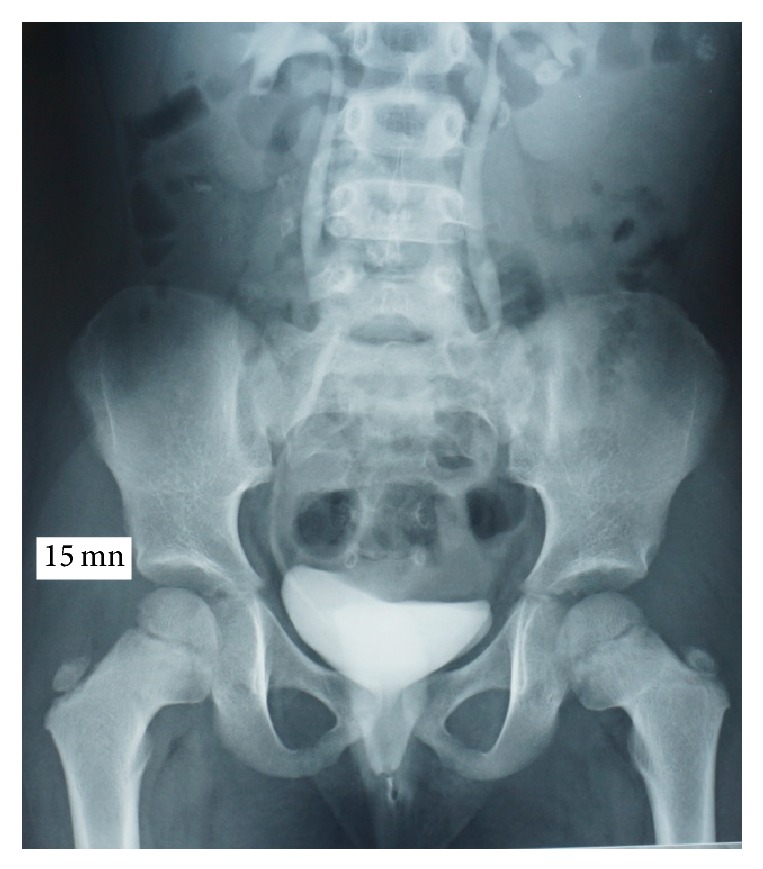
15 min IVU radiography. Bladder filling and vagina opacification.

**Figure 3:**
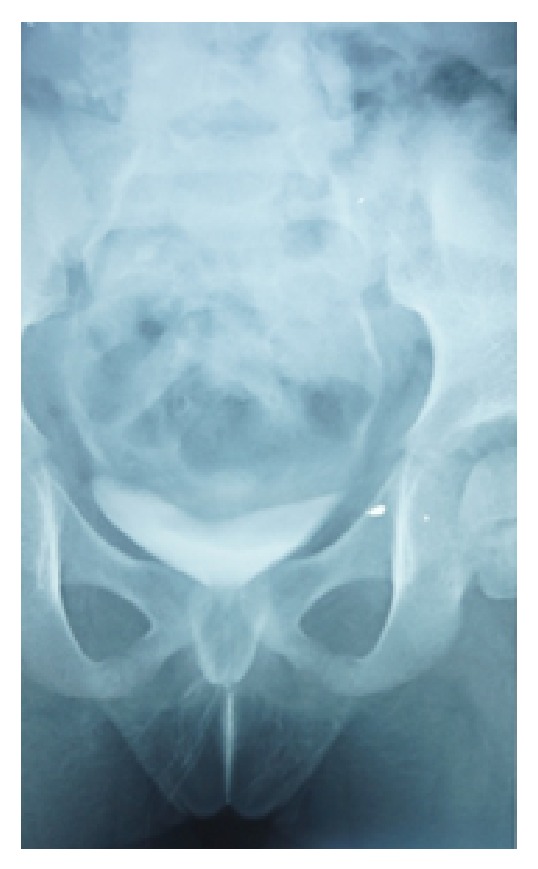
Urinary leakage.

**Figure 4:**
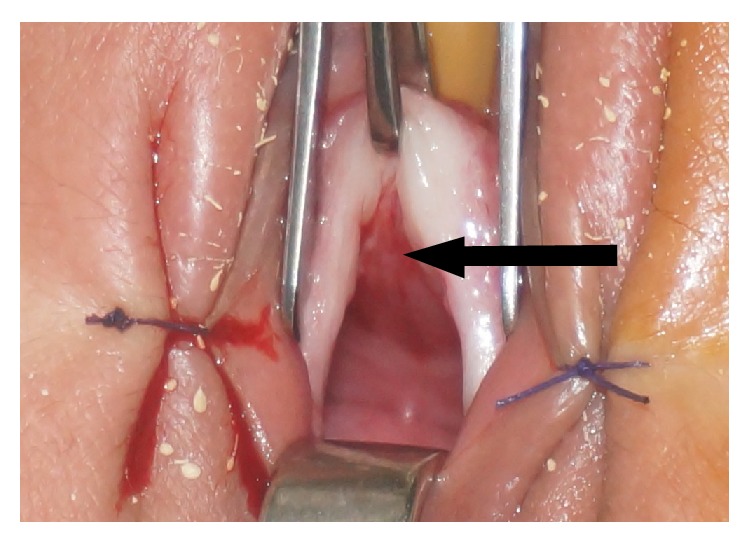
Urethrovaginal fistula difficult to see.

**Figure 5:**
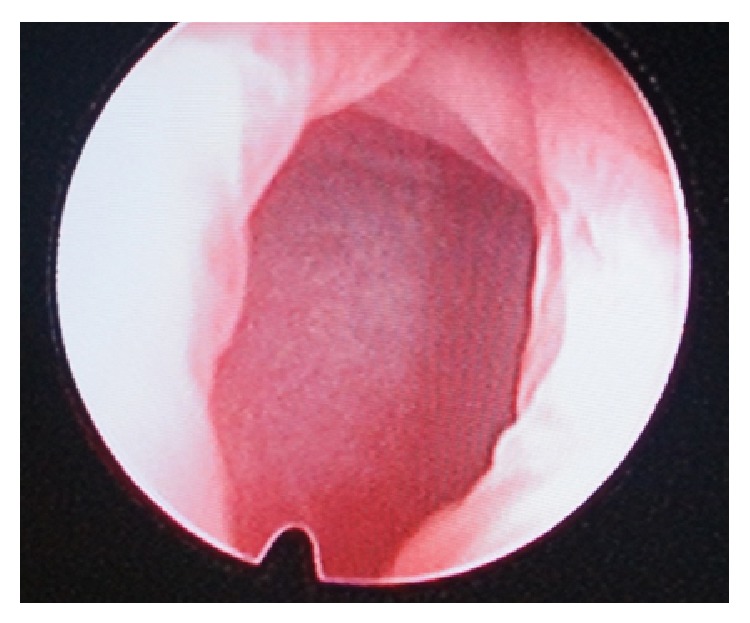
Endoscopic view of fistula.

**Figure 6:**
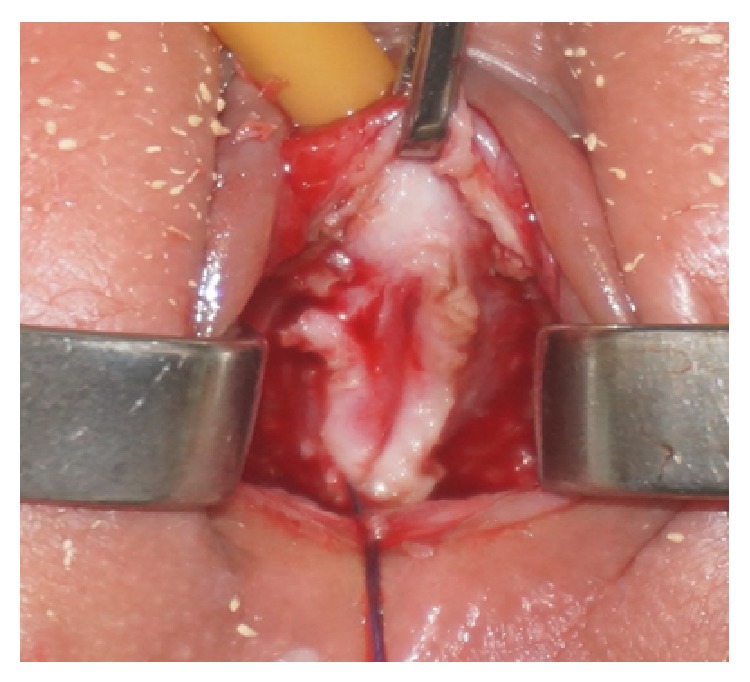
Dissection between urethra and vagina and beginning of urethral closure.

At intravenous urography (IVU) there was no pyelocaliceal expansion. We noted opacification of the vagina by the contrast medium and urine leakage while standing (Figures 2 and 3). An exploration under narcosis was performed and there was a linear urethral fistula (Figure 4). A probe introduced by transurethral route was found in the vagina confirming the fistula. At cystoscopy, a urethral fistula was visible; it was linear and was estimated to be about one centimeter and a half (Figure 5). The bladder was normal with the ureteral meatus in normal position. The cure of the fistula was performed through an incision around the fistula followed by dissection between vagina and urethra (Figure 6). Each structure was then sutured separately. The postoperative course was uneventful.

## 3. Discussion

Urogenital fistulas are a scourge in our working conditions. It is a tragedy for patients suffering from them. They are common in underdeveloped countries and are mainly obstetric [1]. Urethrovaginal fistula occurs mostly in obstetric context or is iatrogenic due to surgical procedures in adults [2, 3]. In girls, it often occurs in a pelvic trauma context [4].

Involuntary loss of urine in a child first evokes enuresis. It may be a congenital etiology when it occurs at birth. When this condition is established, a traumatic etiology is at issue. In the case reported, the scarcity of urethral damage in females and acquired character made us suggest a probable sexual assault that we were not able to confirm. If this were the case, medicolegal aspects should be taken into account [1]. Indeed, a sexual assault itself constitutes a matter of complaint, but the consequences of the treatment may also lead to complaints against the surgeon. This is important to keep in mind as the consequences of fistula and/or its treatment may have an impact on the quality of sexual life of the patient. We found the concept of removal of the bladder catheter, with an inflated balloon, in favor of an iatrogenic etiology. The urethral trauma is in fact the most common etiology. It occurs most often in a pelvic trauma context [5, 6]. In our patient we discussed trauma associated with a urinary catheter. The tube was withdrawn with the inflated balloon according to the parents. It seems difficult to do so without causing major damage. We believe that the balloon could have been incompletely deflated. It would have easily crossed the bladder neck but would have caused injury to urethral midpart. The fistula could also be the result of an ischemic process due to pressure of the catheter on the urethra leading to a pressure sore [7]. Finally, the first surgical procedure could also be involved in the occurrence of the urethral defect. We have no information about this operation. This situation is quite frequent in our condition as the transmission of medical information is not as good as it should be.

Urethrovaginal fistula identification is usually easy [3], but a clinical examination in a young girl may be difficult and delicate. It is impossible to use regular procedures such as “three-sponge” test or blue dye test [1, 8]. We immediately prescribed intravenous urography (IVU) to seek a possible ureteral injury and appreciate the impact on the upper urinary tract. The detailed assessment of the fistula was done under general anesthesia before performing the surgery. We have also made an intraoperative urethrocystoscopy. This enabled us to do the inventory as was the case with other authors [3, 9].

Female urethral lesions are rare and a cause of major cure issues [9, 10]. Little is known about fistula cure in children. It is therefore logical to refer to techniques used in adult patient to manage this condition. The principles of vesicovaginal fistula cure may be applied to urethrovaginal fistula. Key points are excision of the fistula edge and separate closure of each layer. The main issue is exposure. One may use an abdominal approach or a vaginal approach. In the latter one, the patient is installed in lithotomy position or inverted lithotomy position allowing a better access to the fistula [7]. The transanorectal approach is another way to access complex fistulas or fistulas in infants [11]. By this way, the fistula is perfectly accessible and its management is made easier.

Xu series associate urethral stenosis with fistula. Urethrovaginal fistula management difficulties are linked to the risk of urinary incontinence following rehabilitation due to sphincter damage [10].

In our case the location was midurethral. We used a vaginal route with economic tissue resection. The fibrosis around the fistula was relatively easy to dissect. This allowed us to cure the fistula with a satisfactory postoperative result (no micturition disorder due to sphincter damage). In some cases, the surgical strategy is complex. In a case of fistula of the proximal urethra in a 6-year-old girl, the approach was abdominal [5]. This may be explained by fibrosis induced by previous interventions. In our case, the fistula was easily accessible by vaginal route and managed by a single suture without resecting much tissue. Tissue loss is a characteristic of urethrovaginal fistulas. Economic excision of a periurethral tissue is recommended because the narrowness of the operating field does not allow tissue interposition [3]. Our cure was relatively easy as it was the first attempt. The postoperative recovery was simple and immediate and long-term results were considered satisfactory. A cystography 14 days after the procedure is recommended before removing the tube [1]. We do not do cystography in practice. Complications are possible; it may be either urinary incontinence or a urethral stricture to the contrary [3]. These complications are feared and cannot be predicted. Ockrim et al. [2] have found no predictor of success for urethrovaginal fistula cure. In contrast, for a vesicovaginal fistula over 3 cm the absence of tissue interposition is the cause of failure of the treatment. The use of fibrin glue [12] improves outcomes.

## 4. Conclusion

The urethrovaginal fistula is rare in girls. It occurs mainly after a trauma. The circumstances of this trauma should be noted because a forensic aspect should be considered in the case of sexual violence. The treatment is made difficult by lack of tissue available for conventional treatment. In the case reported it was a linear fistula repaired easily with simple suites.
